# Study protocol: evaluation of an online, father-inclusive, universal parenting intervention to reduce child externalising behaviours and improve parenting practices

**DOI:** 10.1186/s40359-017-0188-x

**Published:** 2017-06-19

**Authors:** Lucy A. Tully, Patrycja J. Piotrowska, Daniel A. J. Collins, Kathleen S. Mairet, David J. Hawes, Eva R. Kimonis, Rhoshel K. Lenroot, Caroline Moul, Vicki Anderson, Paul J. Frick, Mark R. Dadds

**Affiliations:** 1 School of Psychology, University of Sydney, Sydney, NSW Australia; 2 School of Psychology, University of New South Wales, Sydney, NSW Australia; 3 School of Psychiatry, Faculty of Medicine, University of New South Wales, Sydney, NSW Australia; 4 Royal Children’s Hospital, Murdoch Children’s Research Institute, Departments of Psychology & Paediatrics, University of Melbourne, Melbourne, VIC Australia; 5 Learning Sciences Institute of Australia, Australian Catholic University, Brisbane, Australia & Department of Psychology, Louisiana State University, Baton Rouge, USA

**Keywords:** Online parenting interventions, Parenting, Fathers, Child externalising problems

## Abstract

**Background:**

Parenting interventions that focus on enhancing the quality and consistency of parenting are effective for preventing and reducing externalising problems in children. There has been a recent shift towards online delivery of parenting interventions in order to increase their reach and impact on the population prevalence of child externalising problems. Parenting interventions have low rates of father participation yet research suggests that father involvement may be critical to the success of the intervention. Despite this, no online parenting interventions have been specifically developed to meet the needs and preferences of fathers, as well as mothers. This paper describes the protocol of a study examining the effectiveness of an online, father-inclusive parenting intervention called ‘ParentWorks’, which will be delivered as a universal intervention to Australian families.

**Methods/design:**

A single group clinical trial will be conducted to examine the effectiveness of ParentWorks for reducing child externalising problems and improving parenting, as well as to explore the impact of father engagement (in two-parent families) on child outcomes. Australian parents/caregivers with a child aged 2–16 years will be recruited. Participants will provide informed consent, complete pre-intervention measures and will then complete the intervention, which consists of five compulsory video modules and three optional modules. The primary outcomes for this study are changes in child externalising behaviour, positive and dysfunctional parenting practices and parental conflict, and the secondary outcome is changes in parental mental health. Demographic information, satisfaction with the intervention, and measures of parental engagement will also be collected. Questionnaire data will be collected at pre-intervention, post-intervention and three-month follow-up, as well as throughout the program.

**Discussion:**

This paper describes the study protocol of a single group clinical trial of a national, online, father-inclusive parenting intervention. The results from this study could be used to inform public policy about providing support to parents of children with behaviour problems, and enhancing the engagement of fathers in parenting interventions.

****Trial registration**:**

ACTRN12616001223426, registered 05/09/2016

## Background

Childhood externalising problems describe behaviour that is characterised by aggression, defiance, hostility and poor impulse control, and these behaviours are the main reason for referral to child and adolescent mental health services [1]. Externalising behaviour problems are associated with a range of social and health difficulties, and at extremes, can lead to a diagnosis of Oppositional Defiant Disorder (ODD), Attention Deficit Hyperactivity Disorder (ADHD) and/or Conduct Disorder (CD), which are collectively known as Disruptive Behaviour Disorders (DBDs). Worldwide prevalence estimates suggest 5.7% of children have ODD or CD and 3.4% have ADHD [2]. Childhood externalising problems and DBDs are among the first reliable signs of emerging social, physical and mental health problems [3, 4] and they are associated with longer-term adverse outcomes such as school dropout, alcohol abuse, poor physical heath and adult psychiatric disorders [4–6]. Fortunately, there is substantial evidence that parenting interventions, which focus on enhancing the quality and consistency of parenting practices, produce lasting improvements in these childhood mental health problems, potentially reducing lifetime burden in at-risk children [7, 8]. In recent years, there has been increasing interest in delivering parenting interventions online via the internet in order to increase dissemination, and initial research indicates that online delivery is effective [9, 10]. Regardless of delivery modality, fathers are consistently underrepresented in parenting interventions [11], yet research indicates greater improvements in parenting and child externalising problems when fathers participate [12]. This paper describes a protocol for an online parenting intervention that has been developed to meet the needs and preferences of fathers, in order to maximise effectiveness.

More than 40 years of research demonstrates that parenting interventions based on social learning theory are effective in decreasing coercive parenting, increasing positive parenting and, in turn, improving child externalising problems [13–16]. Parenting interventions are effective in the short-term and longer-term, with positive effects on child outcomes lasting up to 20 years post-intervention [17]. Parenting interventions can be offered as universal interventions for all parents, or as targeted interventions for more at-risk children and families. Despite the effectiveness of both universal and targeted interventions, research has found that few parents participate in face-to-face parenting interventions and many drop out early. In a recent meta-analytic review of targeted parenting interventions (*k* = 262), Chacko et al. [18] found that at least 25% of parents of children with externalising problems dropped out prior to commencement and 26% dropped out during treatment. Similarly, participation rates are also low in universal parenting interventions. For example, in a randomised controlled trial (RCT) of a universal parenting intervention in Germany, only 31% of the population participated [19]. While there are a number of reasons for low participation rates and high attrition, the practical demands of participation (e.g., time, work, child care, transportation and costs) are likely to be key barriers to participation in traditional face-to-face parenting interventions for many families [20].

In recent years, there has been growing interest in the online delivery of parenting interventions as a way to increase their reach and impact. Online delivery reduces the practical demands of participation, and parents report that they also prefer online delivery to face-to-face sessions [21]. The internet is the resource of choice for many parents to obtain information and advice about parenting [10], including parents of children with mental health problems. An Australian population-based survey found that over one-third of parents of children with mental health problems had used the internet to get help or information about their child’s problems [22]. Video demonstrations of parenting strategies are already an integral component of most evidence-based parenting interventions, so these can be easily included in internet interventions [9]. Online delivery also provides an opportunity to upscale universal parenting interventions and disseminate them widely, which has the potential to impact on population rates of childhood externalising problems [23]. Initial research shows that the effects of online parenting interventions are promising. A meta-analytic review (*k* = 12) found medium effects for parent outcomes and child outcomes [10], which are similar to those found for traditional face-to-face delivery [8].

Online parenting interventions may be entirely self-directed (where parents work through the program without assistance from a practitioner), or accompanied by practitioner guidance or support (via face-to-face assistance, phone or videoconferencing sessions, or email coaching). Most online interventions evaluated to date have included some component of practitioner support, with only four out of 12 studies included in the recent meta-analysis [10] entirely self-directed, although there have been other studies of self-directed interventions that were not included in this review [24]. When considering a public health approach to reduction of child externalising problems, self-directed online interventions have clear benefits such as greater reach, scalability, increased convenience for families, as well as reduced costs for delivery, in terms of not requiring practitioner training or involvement. While not all parents would benefit from an online self-directed intervention, it could be offered as an initial step in a stepped-care approach, with practitioner support subsequently offered to those who require additional assistance [25]. Encouragingly, the only RCT that has compared an online parenting intervention with or without practitioner support found that receiving practitioner support conferred no apparent additional benefits for parent or child outcomes, although the sample size in this study was very small and the focus was on reducing child anxiety [26].

Parenting interventions generally target both mothers and fathers (the core parenting team), but father participation rates are often very low, or not reported at all [11]. Importantly, there is evidence that including fathers in traditional face-to-face parenting interventions leads to improved outcomes. Lundahl et al. [12] conducted a meta-analytic review (*k* = 26) and found that father engagement in parenting interventions was associated with reduced child externalising behaviour and improved parenting behaviour in the short-term, but not in the longer-term. However, other research has found long-term improvements in child outcomes when fathers are included in interventions [27, 28]. Although there are likely to be many reasons for the low rates of father participation, there has been very little research to date about fathers’ perceptions regarding barriers and facilitators to participation [29]. However, three surveys with fathers have found that cost of the service, lack of time and work commitments consistently emerge as key barriers to participation [29–31].

Providing parenting interventions online has the potential to address many of the practical barriers that may prevent fathers from participating in traditional face-to-face interventions. Indeed, there is evidence that fathers prefer online interventions over face-to-face formats [29, 31]. However, like face-to-face interventions, rates of father participation in online parenting interventions are often not reported. Of the 12 papers included in the meta-analysis of online parenting interventions [10], 11 either reported mother results only or did not provide a breakdown of parent gender. This suggests that researchers continue to disregard the importance of both reporting on parent gender and including mothers and fathers in parenting interventions [11]. Consequently, the rates of father engagement in online parenting interventions are unknown. Researchers have highlighted that parenting programs have been designed and tested with mothers, and it may be critical to provide a program that meets the needs and preferences of fathers, in order to achieve high rates of father engagement [32–34].

Surveys of fathers’ needs and preferences have been conducted in order to better tailor the program content, promotion, and delivery of parenting interventions to fathers. The results of these surveys have shown that fathers prefer content focussing on child competencies, such as building positive relationships with children, increasing children’s confidence and social skills [29], and helping children deal with bullying [31]. Fathers have identified several key factors that can influence their decision to participate, including: knowledge of the effectiveness of the program, understanding what is involved in the program, and the facilitator’s level of training [29, 31]. Based on the results of their survey with fathers, Frank et al. [35] adapted a face-to-face parenting intervention to meet the preferences and needs of fathers, leading to high rates of father participation and significant positive changes in father ratings of child behaviour problems and parenting from pre- to post-intervention. Thus, adapting a program to meet the needs and preferences of fathers may enhance engagement and efficacy, yet no online parenting interventions have been developed or adapted with fathers in mind.

In summary, online delivery of parenting interventions has the potential to increase reach and dissemination and impact on child externalising problems. Fathers may be more likely to participate in parenting interventions provided online, especially if they are tailored to meet their needs and preferences. This is critical given that fathers frequently do not participate in typical parenting interventions, despite research indicating their participation is likely to enhance intervention effectiveness. Thus, providing an intervention tailored to the needs and preferences of fathers may enhance both father participation and program outcomes. This paper describes the protocol for a self-directed online parenting intervention called ParentWorks, which has been adapted to meet the needs and preferences of Australian fathers, and is designed to reduce child externalising behaviours and improve parenting. This population-level universal intervention is part of the *Like Father Like Son* project, and is one of several innovative national strategies aimed at enhancing engagement of fathers in evidence-based interventions for childhood externalising problems.

### Objective

This paper describes the protocol for a quasi-experimental repeated measures study that examines the effectiveness of a universal, online parenting intervention called ParentWorks, in reducing father- and mother-reported child externalising behaviour, dysfunctional parenting and parental conflict, and increasing positive parenting. The key research questions include:

1. Does participation in the online parenting program significantly reduce father- and mother- reported dysfunctional parenting, parenting conflict and child externalising behaviour and increase positive parenting from pre- to post-intervention, and are these changes maintained at three-month follow-up?
2. Does participation of fathers (in two-parent families) enhance the outcomes of the intervention in terms of reductions in mother-reported child behaviour problems?
3. What variables predict father engagement in the online parenting program (including socio-demographic variables such as father age and education, and family variables such as parenting conflict and relationship satisfaction)?
4. What are the moderators and mediators of the effectiveness of the online parenting program?

## Methods/Design

### Design

This study is an uncontrolled, single group clinical trial, involving a quasi-experimental, repeated measures design with three assessments (pre-intervention, post-intervention and three-month follow-up). After reading and signing an online consent form, participating parents will complete a pre-intervention questionnaire. Participants will complete the program at their own pace, and then complete a post-intervention questionnaire. Three months after completing the post-intervention questionnaire, they will complete a follow-up questionnaire. The study has been approved by the University of Sydney Human Research Ethics Committee (Project No. 2016/452) and is registered with the Australian New Zealand Clinical Trials Registry (ACTRN12616001223426).

### Participants

Eligible participants will be Australian parents or caregivers of children aged 2–16 years. We aim to recruit 1200 parents/caregivers to participate in the study. To be eligible for the study, participants must be: a parent or caregiver of a child aged 2–16 years; aged 18 and over; currently living in Australia; and able to complete the questionnaires and understand the program content in English. The ParentWorks website will be geoblocked so that only participants located in Australia will be able to register for, and participate in, the program.

### Recruitment of study population

A national media campaign will be conducted to promote ParentWorks through online and social media channels, as well as traditional media formats, such as radio. In order to achieve high rates of father participation, the media campaign will include short videos of fathers talking about the challenges of parenting, and will prompt parents to participate in ParentWorks. In addition to the media campaign, potential participants may also hear about the program through word of mouth, flyers distributed through child and family services, and practitioner recommendations. Interested participants will be directed to the program website for more information and to enrol in the program. While the program website will be specifically developed to be appealing for fathers/male caregivers, mothers will also be encouraged to complete the program, and two-parent families will be encouraged to complete the program together.

**Fig. 1:**
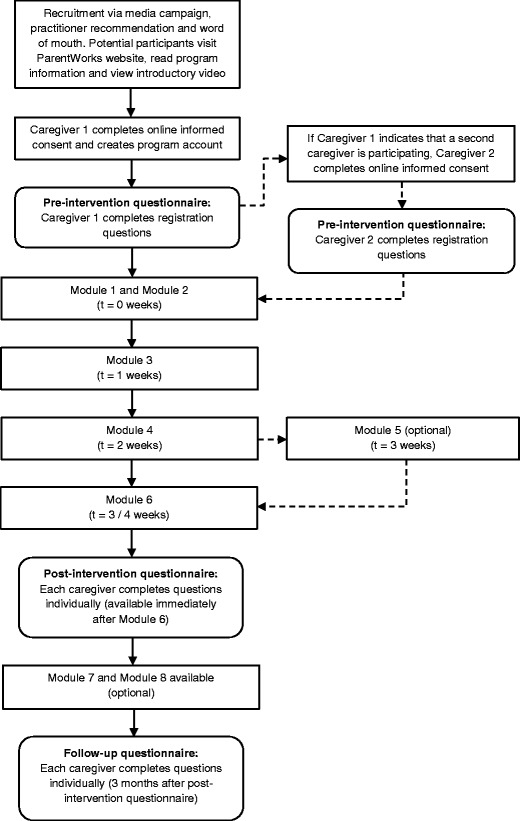
Recruitment and flow of participants through ParentWorks program

Potential participants can watch an introductory video about the program on the ParentWorks website. If they elect to participate in the program, they will be required to read the participant information statement and indicate consent to the conditions listed in the online consent form. As the program will be delivered online, participants will not provide written consent, but will indicate their consent to participate by clicking a box acknowledging that they have read the information statement and they agree to participate. They will then register for the program by completing a series of questions in an online registration form. The questionnaires will be anonymous and no identifying information will be obtained. There will be a separate registration for each parent/caregiver (for two-parent families), and each parent will complete questionnaires independently throughout the program. The parent who initially registers for the program is encouraged to discuss program participation with their partner/co-parent before making a decision about whether to participate in the program with or without their partner/co-parent. See Fig. 1 for a flowchart of participant recruitment and progression through the program.

### Intervention

ParentWorks is based on the *Integrated Family Intervention for Child Conduct Problems* developed by Professor Mark Dadds and Associate Professor David Hawes [36]. Previous research studies have evaluated this intervention in different delivery formats including face-to-face delivery [37] and a web-based version that included videoconferencing sessions with a practitioner [38, 39]. This program has been found to be effective in reducing child externalising problems in both formats. This intervention was originally developed only for parents of children with conduct problems, so it has been modified for ParentWorks to be suitable for a broader community sample of parents who may have more general concerns about parenting and child behaviour. It has also been adapted for self-directed delivery, with no practitioner involvement.

ParentWorks can be completed via the internet using a computer, tablet, or mobile phone. The program comprises video presentations of eight interactive sequenced ‘modules’, five of which are compulsory. Each video module is approximately 20–30 min in duration. The module content is described in detail in Table 1, and sequencing of modules is depicted in Fig. 1. Participants work through the program at their own pace, and depending on which modules they choose, they are able to complete the program in a minimum of three weeks (or four weeks, if they elect to complete Module 5). In order to maximise program flexibility, participants can take as long as they like to complete the program, and can also put it on hold for as long as they choose. Modules 1 and 2 can be completed together immediately after the pre-intervention questionnaire. Modules 3–6 are unlocked sequentially one week after completing the previous module, to allow time for parents to implement the program strategies between modules. This is similar to the format for face-to-face programs, and prevents parents moving through the program too quickly, without practicing the key skills. Parents elect whether or not to complete Module 5 on the topic of *Working as a Team* (see Table 1). Based on pre-questionnaire responses concerning parenting conflict (see Measures section), participants may receive a recommendation to complete this module. After finishing Module 6 (*Review and Preventing Future Problems*), parents complete post-intervention questionnaires and may download a completion certificate. Thus, participating parents watch either five or six modules prior to completion of post-intervention questionnaires. They can then elect to complete optional Modules 7 and 8 on the topics of *Encouraging Child Development Through Quality Time and Play* and *Bully-Proofing Your Child* (see Table 1). These modules were specifically added based on the results of survey research with fathers, which indicated their preferences for content on these topics [31].

**Table 1:** Description of ParentWorks module content

Module	Content	Duration	Compulsory/Optional	Homework exercises
Module 1: Getting Started and Setting Up for Success	•Discussion about how the program works •Causes of child behaviour •The important role that fathers play in their children’s upbringing •Barriers to completing the program and how to overcome them •Setting goals for the program	17 min	Compulsory	No
Module 2: Encouraging Positive Behaviour	•The importance of when and how parents give children attention •Introducing the concept of attachment-rich interactions with children and how children may meet their attachment needs through both good behaviour and misbehaviour •The importance of parents spending quality time with their children •Information about the different strategies to reward children for positive behaviour, including descriptive praise, tangible rewards, physical affection and spending quality time with them	21 min	Compulsory	Yes
Module 3: Responding to Misbehaviour	•Information about the following strategies: setting family rules, giving instructions, using time-out •Trouble-shooting tips for using time-out •Downloadable tip sheet on the topic of Discipline Strategies for Older Children and Teens	26 min	Compulsory	Yes
Module 4: Managing Challenging Situations and Sibling Conflict	•Information about high-risk situations (e.g., car rides, morning routines and going shopping with children) •Practical strategies to manage high-risk situations using step-by-step instructions on what to do before, during and after an event •Practical strategies to manage sibling conflict, such as rewarding and disciplining siblings as a team	25 min	Compulsory	Yes
Module 5: Working as a Team	•Common sources of disagreement between parents •Practical advice for parents about: 1. what to do when their child misbehaves whilst two adults are present; 2. how to have brief discussions together; 3. problem-solving discussions •The importance of spending quality time together and practical ways to achieve this •Advice for separated/divorced parents •Downloadable tip sheet on Co-Parenting Tips for Separated and Divorced Parents	13 min	Optional- If not selected immediately after Module 4, this module is available for completion after post-intervention questionnaire	Yes
Module 6: Review and Preventing Future Problems	•Information presented on key strategies to maintain changes •Summary of the key points and strategies provided in modules 2 to 5 •Reminder that they have the option to complete the additional modules and download the tip sheets available •Parents prompted to complete the post-intervention questionnaire and reminded that they will then receive feedback on their progress •Parents informed that they will receive an email reminder to complete the three-month follow-up questionnaire	15 min	Compulsory	Yes
Bonus Tip Sheets	Downloadable tip sheets on: •Managing Children’s Worry and Low Mood •Setting up Good Sleep Habits for Children •Improving Children’s Social Skills •Setting Healthy Limits on Screen Time	N/A	Optional – available after post-intervention questionnaire	N/A
Module 7: Encouraging Child Development through Quality Time and Play	•Information about spending quality time with children •The developmental benefits of playing with children (e.g., cognitive, social, emotional and physical) •Information and practical strategies for enacting child-directed play	19 min	Optional – completed after post-intervention questionnaire	Yes
Module 8: Bully-Proofing Your Child	•Definition of the various forms of bullying (e.g., verbal, psychological and social) •The effects of bullying on the victim, bully and witnesses •Strategies parents can use to reduce the chances of their child being bullied and what they can do if their child is being bullied •How to carry out problem solving discussions with children if they disclose that they are being bullied •Strategies parents can use to reduce the likelihood that their child will bully others •Signs that may indicate that their child is bullying others, why children might bully others as well as what parents can do if their child is bullying others	21 min	Optional – completed after post-intervention questionnaire	Yes

As the intervention does not include any direct assistance from a practitioner, a brief motivational interviewing component has been incorporated into Module 1 with the aim of increasing motivation and engagement in the intervention. Previous research in face-to-face interventions has demonstrated that additional content based on motivational interviewing may enhance motivation, engagement and adherence to parenting interventions [40]. In addition, this motivational interviewing component is designed to encourage parents to self-reflect on their plans for changing their parenting and readiness to complete the online program. Parents will be prompted to consider a range of potential barriers to participation and reflect on strategies for overcoming these barriers.

The ParentWorks website and program content has been designed to appeal specifically to fathers. As it is a free, online intervention that can be completed by parents at home and in their own time, ParentWorks addresses several key barriers to father participation identified in previous research including: cost of service, lack of time, and work commitments [29–31]. Based on survey data, it appears that fathers are particularly concerned with knowing about program content, effectiveness and the facilitator’s level of training [29, 31], therefore this information has been highlighted on the public pages of the website so that fathers are well-informed prior to participation. ParentWorks includes a male clinical psychologist presenting the material (speaking to camera or as a voiceover) and role-plays of fathers (and mothers) demonstrating the main strategies.

As well as video content, the program includes bullet points during the videos that summarise the main ideas presented, in-session exercises/worksheets (approximately three per module), and downloadable homework sheets (‘putting it into practice’ exercises). The text that participants enter into the in-session worksheets also appears in an online workbook, along with summaries of module content, which can be viewed and downloaded after each module is completed (the information recorded in the in-session worksheet will not be included in the study dataset). As shown in Table 1, there are additional tip sheets that parents can download during the program and after completing the post-intervention questionnaires. Similar to other online parenting interventions [24], the focus of ParentWorks is on promoting parental self-regulation and self-monitoring by encouraging parents to set goals after each session, implement positive parenting strategies between sessions, review their implementation of strategies, problem-solve difficulties that arise, and set further goals.

The program includes a number of innovative features to ensure it is user-friendly, personalised and flexible. Firstly, participating parents will receive tailored feedback in the form of automated assessment summaries of questionnaire results on child behaviour, parenting conflict and their own mood in a section called ‘My Family Feedback’. This feedback will be provided at the start and conclusion of the program, and again three months later. If scores are within the high range for any of these measures at any of the time points, participants will be directed to a ‘Resources and Help’ page with links to relevant community services that can provide them with more specialised assistance. Secondly, participants will complete questions within each module about their parenting confidence and their child’s behaviour over the previous week, and these ratings will appear progressively on two ‘Track My Progress’ graphs, to illustrate changes over the course of the program. Thirdly, each participating parent will receive email prompts to increase the likelihood of program completion. Email reminders will be sent in the following cases: incomplete registration; the next module is unlocked; post-intervention or follow-up questionnaires have not been completed; the program has not been accessed for three weeks; and the program has been on hold for four weeks. Finally, to track engagement of both parents (in two-parent families), parents will also be asked to identify who is watching each module. Once a module has been viewed, it can be re-watched as many times as a participant wishes, and (for two-parent families) parents will be asked who is watching upon each repeat viewing. The program automatically records the number of times each module is watched and the viewing date, so time taken to complete the program can be tracked.

As part of the development of ParentWorks, the website underwent usability testing with a sample of 100 parents, 54 of whom were fathers. This aimed to determine acceptability of the site to parents, and to ensure that it appealed to fathers. Two thirds (66%) of participants rated the website as either very good or excellent overall, and 91% thought the website was suitable for fathers.

### Measures

The following primary outcome measures will be completed as self-report scales by parents/caregivers (both mothers and fathers) at pre-intervention, post-intervention and three-month follow-up assessment to evaluate the effectiveness of the online parenting program:

1. *Strengths and Difficulties Questionnaire* (SDQ; [41]) will measure child emotional/behavioural adjustment. The total difficulties score will be used to measure child emotional and/or behavioural problems; the conduct problems and hyperactivity subscales will be used specifically to measure externalising behaviour problems.
2. *Parent Problem Checklist* (PPC; [42]) problem score will measure disagreements between parents over childrearing issues in two-parent families only.
3. Two subscales from the *Parenting and Family Adjustment Scales* (PAFAS; [43]) will be used to measure both dysfunctional and positive parenting.

For completion of the SDQ, caregivers with more than one child will be asked to select a ‘target’ child to answer these questions about. The target child is the child aged 2–16 whose behaviour or development the parent/caregiver is most concerned about, or, if they have no concerns about their children, the youngest child within this age range. The first caregiver to register for the program will select this target child, and to ensure both caregivers are answering questions about the same child, the child’s name will then be used in the questionnaires completed by the second caregiver (however, this name will not be included in the study dataset).

The K6 [44] will be used as a secondary outcome measure to measure parental mental health at pre-intervention, post-intervention and follow-up assessment.

At pre-intervention, all participating parents will complete a range of socio-demographic questions about themselves and their families, and information about the target child (such as age, diagnosis of mental health problems, and previous assistance for child’s emotions, behaviour or development). Further questions were developed specifically for the study to assess: amount of time co-parents spent discussing program content (at post-intervention only); level of perceived involvement of parent in child’s life (pre-intervention only); level of chaos in home environment (pre- and post-intervention); and whether any additional assistance had been sought since commencing the program (post-intervention only). Satisfaction with the program will be assessed at post-intervention with five items from Eyberg’s *Therapy Attitude Inventory* [45].

Participating caregivers will be asked a range of questions which are built into each video module. If two caregivers are registered for the program, they will first be asked to select who is watching the module and subsequent questions will then be directed only to those caregivers who are viewing the module (although names will not be included in the study dataset). At the start of each module, caregivers will be asked to rate their child’s behaviour (on a 10-point scale from ‘no behaviour problems’ to ‘significant problems’) and their parenting confidence over the previous week (on a 10-point scale from ‘not at all confident’ to ‘extremely confident’). From module 3 onwards, they will also be asked the extent to which they used the strategies from the previous module, on a 10-point scale from ‘not at all’ through to ‘frequently’. At the end of each module, caregivers will rate the module content on a 7-point scale from ‘not at all helpful’ through to ‘extremely helpful’.

### Data collection procedure

Information from participating parents/caregivers will be collected through online questionnaires completed at three time points (as detailed above) with email reminders sent up to three times to ensure high rates of questionnaire completion. Weekly data about parent confidence, child behaviour and use of program strategies will also be collected, as well as number of times each module is viewed. No identifying information will be included in the dataset. Data will be periodically downloaded to the University of Sydney server and stored on a password-protected computer drive accessible only to project staff based at the University.

### Data analysis procedure

Analyses of changes from pre- to post-intervention and at three-month follow-up on the primary and secondary outcome measures for mothers and fathers will be conducted using repeated measures univariate and multivariate analyses. Propensity score matching will be used to examine the overall intervention effects accounting for the bias of family structure (e.g., father involvement in two-parent families). To examine the effect of level of father involvement in the program (in two-parent families) on child outcomes (as rated by mothers), regression-based analyses will be used. Similarly, regression-based analyses will also be used to examine the predictors of father engagement and the moderators and mediators of the intervention effects. Data will be analysed using SPSS Statistics 22 and M*plus*. The plan for the management of missing data will be informed by missing data analyses used to determine whether data is missing at random or not. If the analysis indicates the missing data is not random, or missing data is extensive, multiple imputation procedures will be used.

### Sample size

The sample size calculation was conducted with G*Power [46]. To detect a small effect of ParentWorks on the primary measure of child outcomes (SDQ; Goodman, 2007) with *d* = 0.2 and 0.8 power, a final sample size of 800 two-parent families is required. Of these families, we expect that approximately 400 fathers will elect to participate. In order to account for attrition rates, and participation of single-parent families, we aim to recruit at least 1200 families. This sample size will also be sufficient to conduct the analyses examining level of father involvement, predictors, moderators, and mediators. There are no plans to carry out interim analyses.

### Reporting of results

Reporting of the trial will follow the TREND statement for behavioural and public health interventions involving non-randomised designs [47]. The overall trial results will be communicated through presentations at national and international conferences, and articles in peer-reviewed scientific journals. It is a requirement of the trial funding body that the results are reported in open-access, peer-reviewed publications.

## Discussion

Online parenting interventions have the potential to address many of the practical barriers to participation in traditional face-to-face interventions, especially for fathers. Currently, fathers are significantly underrepresented in research on evidence-based parenting interventions, including those delivered online. Thus, the online parenting intervention described in the present study protocol is specifically developed to meet the needs and preferences of fathers, as well as mothers. By considering fathers’ needs and preferences in regard to program content, and targeting the intervention to fathers as well as mothers, it is expected that both father engagement and the effectiveness of the intervention will be maximised. This study has a number of additional strengths. Firstly, by collecting and evaluating data from all participating parents, it will contribute valuable information about intervention effectiveness using both mothers’ and fathers’ ratings of parenting and child behaviour, and will allow for the investigation of any additional benefits due to father participation. Secondly, this will be one of only a few studies conducted to examine a universal online parenting intervention provided without any practitioner support, and will thus add to the evidence base about the effectiveness of self-directed interventions. Third, the inclusion of a three-month follow-up evaluation will allow for examination of whether changes in child behaviour and parenting are maintained over time. Finally, this study will use a large-scale media campaign to support the recruitment of a large sample size, which has not yet been achieved in similar research to date.

Despite these strengths, the use of a single group repeated measures design is a methodological weakness, which results in the inability to control for potential confounds, such as maturation effects in children. However, given that this study involves dissemination of a universal parenting intervention, with recruitment supported by a national media campaign, it would not be feasible to implement it as part of a randomised controlled trial. While this design limitation will temper conclusions regarding the effectiveness of the intervention, the strengths of this study have the potential to contribute significant knowledge and inform public policy about enhancing the engagement of fathers in parenting interventions, implementing universal online parenting interventions, and establishing a national approach to reducing child externalising problems.

## Abbreviations

ADHD: Attention deficit hyperactivity disorder
CD: Conduct disorder
DBD: Disruptive behaviour disorder
ODD: Oppositional defiant disorder
PAFAS: Parenting and family adjustment scales
PPC: Parent problem checklist
SDQ: Strengths and difficulties questionnaire

## References

[1] Kazdin AE. Conduct disorder in childhood and adolescence. 2nd ed. Thousand Oaks, CA: Sage; 1995.

[2] Polanczyk GV, Salum GA, Sugaya LS, Caye A, Rohde LA (2015). Annual research review: a meta‐analysis of the worldwide prevalence of mental disorders in children and adolescents. J Child Psychol Psychiatry.

[3] Copeland WE, Shanahan L, Costello EJ, Angold A (2009). Childhood and adolescent psychiatric disorders as predictors of young adult disorders. Arch Gen Psychiatry.

[4] Kim-Cohen J, Caspi A, Moffitt TE, Harrington H, Milne BJ, Poulton R (2003). Prior juvenile diagnoses in adults with mental disorder: developmental follow-back of a prospective-longitudinal cohort. Arch Gen Psychiatry.

[5] Colman I, Murray J, Abbott RA, Maughan B, Kuh D, Croudace TJ, Jones PB (2009). Outcomes of conduct problems in adolescence: 40 year follow-up of national cohort. BMJ.

[6] Fergusson DM, John Horwood L, Ridder EM (2005). Show me the child at seven: the consequences of conduct problems in childhood for psychosocial functioning in adulthood. J Child Psychol Psychiatry.

[7] Dretzke J, Davenport C, Frew E, Barlow J, Stewart-Brown S, Bayliss S, Taylor RS, Sandercock J, Hyde C (2009). The clinical effectiveness of different parenting programmes for children with conduct problems: a systematic review of randomised controlled trials. Child Adolesc Psychiatry Ment Health.

[8] Sanders MR, Kirby JN, Tellegen CL, Day JJ (2014). The triple P-positive parenting program: a systematic review and meta-analysis of a multi-level system of parenting support. Clin Psychol Rev.

[9] Breitenstein SM, Gross D, Christophersen R (2014). Digital delivery methods of parenting training interventions: a systematic review. Worldviews Evid Based Nurs.

[10] Nieuwboer CC, Fukkink RG, Hermanns JM (2013). Online programs as tools to improve parenting: a meta-analytic review. Child Youth Serv Rev.

[11] Panter-Brick C, Burgess A, Eggerman M, McAllister F, Pruett K, Leckman JF (2014). Practitioner review: engaging fathers-recommendations for a game change in parenting interventions based on a systematic review of the global evidence. J Child Psychol Psychiatry.

[12] Lundahl BW, Tollefson D, Risser H, Lovejoy MC (2008). A meta-analysis of father involvement in parent training. Res Soc Work Pract.

[13] Barlow J, Smailagic N, Ferriter M, Bennett C, Jones H. Group-based parent-training programmes for improving emotional and behavioural adjustment in children from birth to three years old. Cochrane Database Syst Rev. 2010. doi:10.1002/14651858.CD003680.pub2. 10.1002/14651858.CD003680.pub2 PMC4164454 20238324

[14] Eyberg SM, Nelson MM, Boggs SR (2008). Evidence-based psychosocial treatments for children and adolescents with disruptive behavior. J Clin Child Adolesc Psychol.

[15] Furlong M, McGilloway S, Bywater T, Hutchings J, Smith S, Donnelly M. Behavioral and cognitive-behavioural group-based parenting interventions for early-onset conduct problems in children age 3-12 years. Cochrane Database Syst Rev. 2012. doi:10.1002/14651858.CD008225.pub2. 10.1002/14651858.CD008225.pub2 PMC12935172 22336837

[16] Lundahl BW, Risser HJ, Lovejoy MC (2006). A meta-analysis of parent training: moderators and follow-up effects. Clin Psychol Rev.

[17] Sandler I, Schoenfelder E, Wolchik S, MacKinnon D (2011). Long-term impact of prevention programs to promote effective parenting: lasting effects but uncertain processes. Annu Rev Psychol.

[18] Chacko A, Jensen SA, Lowry LS, Cornwell M, Chimklis A, Chan E, Lee D, Pulgarin B (2016). Engagement in behavioral parent training: review of the literature and implications for practice. Clin Child Fam Psychol Rev.

[19] Heinrichs N, Bertram H, Kuschel A, Hahlweg K (2005). Parent recruitment and retention in a universal prevention program for child behavior and emotional problems: barriers to research and program participation. Prev Sci.

[20] Reardon T, Harvey K, Baranowska M, O’Brien D, Smith L, Creswell C. What do parents perceive are the barriers and facilitators to accessing psychological treatment for mental health problems in children and adolescents? A systematic review of qualitative and quantitative studies. Eur Child Adolesc Psychiatry. 2017;26:623–47. 10.1007/s00787-016-0930-6 PMC5446558 28054223

[21] Metzler CW, Sanders MR, Rusby JC, Crowley RN (2012). Using consumer preference information to increase the reach and impact of media-based parenting interventions in a public health approach to parenting support. Behav Ther.

[22] Lawrence D, Johnson SJH, Boterhoven De Haan K, Sawyer M, Ainley J, Zubrick SR (2015). The mental health of children and adolescents.

[23] Jones DJ, Forehand R, Cuellar J, Kincaid C, Parent J, Fenton N, Goodrum N (2013). Harnessing innovative technologies to advance children’s mental health: behavioral parent training as an example. Clin Psychol Rev.

[24] Sanders MR, Baker S, Turner KM (2012). A randomized controlled trial evaluating the efficacy of triple P online with parents of children with early-onset conduct problems. Behav Res Ther.

[25] Haaga DA (2000). Introduction to the special section on stepped care models in psychotherapy. J Consult Clin Psychol.

[26] Morgan AJ, Rapee RM, Bayer JK (2016). Prevention and early intervention of anxiety problems in young children: a pilot evaluation of cool little kids online. Internet Interv.

[27] Webster-Stratton C (1985). The effects of father involvement in parent training for conduct problem children. J Child Psychol Psychiatry.

[28] Bagner DM, Eyberg SM (2003). Father involvement in parent training: when does it matter?. J Clin Child Adolesc Psychol.

[29] Frank TJ, Keown LJ, Dittman CK, Sanders MR (2015). Using father preference data to increase father engagement in evidence-based parenting programs. J Child Family Stud.

[30] Sanders MR, Dittman CK, Keown LJ, Farruggia SP, Rose D (2010). What are the parenting experiences of fathers? the use of household survey data to inform decisions about the delivery of evidence-based parenting interventions to fathers. Child Psychiatry Hum Dev.

[31] Tully LA, Piotrowska PJ, Collins DJ, Mairet K, Black N, Kimonis ER, Hawes DJ, Moul C, Lenroot R, Frick PJ et al. Optimizing child outcomes from parenting interventions: Fathers’ experiences, preferences and barriers to participation. BMC Public Health. 2017. doi:10.1186/s12889-017-4426-1. 10.1186/s12889-017-4426-1 PMC5463495 28592244

[32] Fabiano GA (2007). Father participation in behavioral parent training for ADHD: review and recommendations for increasing inclusion and engagement. J Fam Psychol.

[33] Fletcher R, Freeman E, Matthey S. The impact of behavioural parent training on fathers’ parenting: A meta-analysis of the Triple P-Positive Parenting Program. Fathering: A Journal of Theory, Research, and Practice about Men as Fathers. 2011;9:291–312.

[34] Tiano JD, McNeil CB. The inclusion of fathers in behavioral parent training: a critical evaluation. Child Fam Behav Ther. 2005;27:1–28. doi:10.1300/J019v27n04_0l.

[35] Frank TJ, Keown LJ, Sanders MR (2015). Enhancing father engagement and interparental teamwork in an evidence-based parenting intervention: a randomized-controlled trial of outcomes and processes. Behav Ther.

[36] Dadds MR, Hawes DJ (2005). Integrated family intervention for child conduct problems.

[37] Hawes DJ, Dadds MR (2005). The treatment of conduct problems in children with callous-unemotional traits. J Consult Clin Psychol.

[38] Kirkman JJ, Hawes DJ, Dadds MR (2016). An open trial for an e-health treatment for child behavior disorders I: social acceptability, engagement, and therapeutic process. Evid Based Pract Child Adoles Mental Health.

[39] Kirkman JJ, Hawes DJ, Dadds MR (2016). An open trial for an e-health treatment for child behavior disorders II: outcomes and clinical implications. Evid Based Pract Child Adoles Mental Health.

[40] Nock MK, Kazdin AE (2005). Randomized controlled trial of a brief intervention for increasing participation in parent management training. J Consult Clin Psychol.

[41] Goodman R (1997). The strengths and difficulties questionnaire: a research note. J Child Psychol Psychiatry.

[42] Dadds MR, Powell MB (1991). The relationship of interparental conflict and global marital adjustment to aggression, anxiety, and immaturity in aggressive and nonclinic children. J Abnorm Child Psychol.

[43] Sanders MR, Morawska A, Haslam DM, Filus A, Fletcher R (2014). Parenting and family adjustment scales (PAFAS): validation of a brief parent-report measure for use in assessment of parenting skills and family relationships. Child Psychiatry Hum Dev.

[44] Kessler RC, Andrews G, Colpe LJ, Hiripi E, Mroczek DK, Normand S-L, Walters EE, Zaslavsky AM (2002). Short screening scales to monitor population prevalences and trends in non-specific psychological distress. Psychol Med.

[45] Eyberg SM. Consumer satisfaction measures for assessing parent training programs. In: VandeCreek L, Knapp S, Jackson TL, editors. Innovations in clinical practice: a source book. Vol. 12. Sarasota: Professional Resource Press; 1993. p. 377–382.

[46] Faul F, Erdfelder E, Lang A-G, Buchner A (2007). G* power 3: a flexible statistical power analysis program for the social, behavioral, and biomedical sciences. Behav Res Methods.

[47] Des Jarlais DC, Lyles C, Crepaz N (2004). Improving the reporting quality of nonrandomized evaluations of behavioral and public health interventions: the TREND statement. Am J Public Health.

